# Ginsenoside Rg-1 Protects Retinal Pigment Epithelium (RPE) Cells from Cobalt Chloride (CoCl_2_) and Hypoxia Assaults

**DOI:** 10.1371/journal.pone.0084171

**Published:** 2013-12-27

**Authors:** Ke-ran Li, Zhi-qing Zhang, Jin Yao, Yu-xia Zhao, Jing Duan, Cong Cao, Qin Jiang

**Affiliations:** 1 Department of Eye, the Affiliated Eye Hospital of Nanjing Medical University, Nanjing, Jiangsu, China; 2 Institute of Neuroscience, Soochow University, Suzhou, Jiangsu, China; 3 The Center for Safety Evaluation of Drugs, Academic Institute of Pharmaceutical Science, China Pharmaceutical University, Nanjing, Jiangsu, China; Rutgers University, United States of America

## Abstract

Severe retinal ischemia causes persistent visual impairments in eye diseases. Retinal pigment epithelium (RPE) cells are located near the choroidal capillaries, and are easily affected by ischemic or hypoxia. Ginsenoside Rg-1 has shown significant neuroprotective effects. This study was performed to test the cytoprotective effect of ginsenoside Rg-1 in RPE cells against hypoxia and cobalt chloride (CoCl_2_) assaults, and to understand the underlying mechanisms. We found that Rg-1 pre-administration significantly inhibited CoCl_2_- and hypoxia-induced RPE cell death and apoptosis. Reactive oxygen specisis (ROS)-dependent p38 and c-Jun NH(2)-terminal kinases (JNK) MAPK activation was required for CoCl_2_-induced RPE cell death, and Rg-1 pre-treatment significantly inhibited ROS production and following p38/JNK activation. Further, CoCl_2_ suppressed pro-survival mTOR complex 1 (mTORC1) activation in RPE cells through activating of AMP-activated protein kinase (AMPK), while Rg-1 restored mTORC1 activity through inhibiting AMPK activation. CoCl_2_-induced AMPK activation was also dependent on ROS production, and anti-oxidant N-acetylcysteine (NAC) prevented AMPK activation and RPE cell death by CoCl_2_. Our results indicated that Rg-1 could be further investigated as a novel cell-protective agent for retinal ischemia.

## Introduction

In the pathology of central retinal vein occlusion (CRVO), age-related macular degeneration (AMD), diabetic retinopathy (DR) and retinopathy of prematurity (ROP), persistent retinal ischemia causes severe visual impairments, which will lead to blindness if not handled appropriately [1]. An interruption in blood supply to the retina will cause tissue ischemia, which leads to hypoxia and a rapid failure of energy production, and subsequent cell apoptosis or necrosis [1]. Several animal and cell models have been utilized to study retinal ischemia. Meanwhile, an increasing number of agents have been tested to interrupt the ischemic-hypoxia cascade, and to slow down or even reverse the retinal ischemia progression [2].

Ginseng is a well-known Chinese traditional medicine, and has shown significant anti-oxidative and pro-cell survival abilities [3]. It can also regulate intracellular calcium homeostasis in treating diabetes and cardiovascular disease [3], [4], [5]. It is safe and nontoxic to both animals and human even at high doses. Ginsenosides are the pharmacologically active components of ginseng. Among nearly 40 different ginsenosides isolated from ginseng, Ginsenoside Rg-1 is known as the major active component responsible for many pharmaceutical actions of ginseng [6], [7], [8]. Recent studies have shown that Rg-1 possesses significant neurotrophic and neuroprotective effects against stresses including hydrogen peroxide [9], β-amyloid [10], [11], [12], glutamate [6], [13], [14], 1-methyl-4-phenylpyridinium (MPP^+^) [15] and rotenone [16]. *In vivo* studies also demonstrated that Rg-1 is neuron protective in rat models of Parkinson's disease (PD) [17], [18], [19], Alzheimer's disease (AD) [20], [21] and hypoxic-ischemic injuries [22]. In addition, Rg-1's anti-inflammatory activities have also been shown by many groups. Rg-1 inhibits immune cells activation and subsequent release of pro-inflammatory cytokines, through preventing the activation of transcriptional factors NF-KB and MAPK [23], [24], [25]. Thus, ginsenoside Rg-1 is an ideal cell-protective candidate.

The retinal pigment epithelium (RPE) is the pigmented cell layer just outside the neurosensory retina that nourishes retinal visual cells, and is firmly attached to the underlying choroid and overlying retinal visual cells [26]. The RPE cells are located close to the choroidal capillaries, and are easily to be affected in an ischemic or hypoxia condition [27]. Considering that RPE cells are also neuron origin, in the current study, we studied the potential protective effect of Rg-1 against cobalt chloride (CoCl_2_, chemical hypoxia) and hypoxia assaults in cultured RPE cell, and to investigate the underlying mechanisms.

## Results

### Rg-1 suppresses CoCl_2_-induced RPE cell death

**Figure 1:**
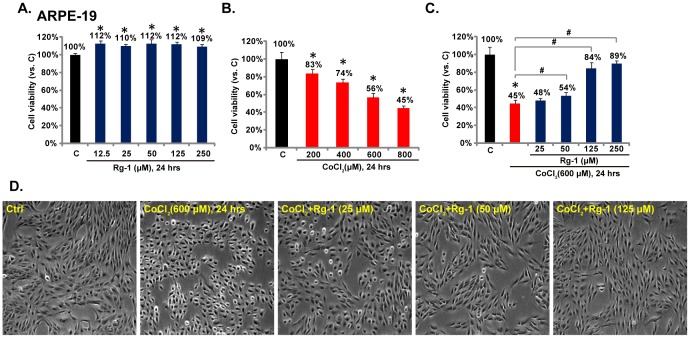
Rg-1 suppresses CoCl_2_-induced RPE cell death. ARPE-19 cells were treated with indicated concentration of Rg-1 (A) or CoCl_2_ (B) for 24 hrs, cell viability was analyzed by MTT assay. ARPE-19 cells were pre-treated with indicated concentration of Rg-1 (25, 50, 125 and 250 µM) for 2 hrs, followed by CoCl_2_ (600 µM) administration, cells were further cultured for 24 hrs, and cell viability was tested (C), cell morphology was also presented (D).Data were expressed as mean±SD. Experiments were repeated three times, and similar results were obtained (same for all figures). *p<0.05 vs. group “C” (Control). **^#^** ***p***<0.05.

Cell viability results in Figure 1A showed that Rg-1 by itself showed no cytotoxicity against RPE cells even at a high dose. As a matter of fact, it slightly increased RPE cell survival (Figure 1A). CoCl_2_-induced cytotoxicity in RPE cells was also tested by the MTT assay, and results in Figure 1B showed that CoCl_2_ stimulation at different concentrations (0, 200, 400, 600 and 800 µM, 24 hrs) caused significantly RPE cell viability reduction (cell death). The effect of CoCl_2_ was dose-dependent. 600 µM CoCl_2_ caused an approximate 50% reduction in RPE cell viability, and this concentration was set to induce RPE cell damage in the subsequent experiments (Figure 1B). As shown in Figure 1C, pretreatment with Rg-1 at concentrations of 50, 125 and 250 µM significantly inhibited CoCl_2_-induced reduction of cell viability, 25 µM of Rg-1 pre-administration also slightly suppressed RPE cell death by CoCl_2_, but the difference was not significant (Figure 1C). Cell morphological changes were consistent with the protective effect of Rg-1 against CoCl_2_ in RPE cells (Figure 1D).

### Rg-1 inhibits CoCl_2_-induced apoptosis of RPE cells

**Figure 2:**
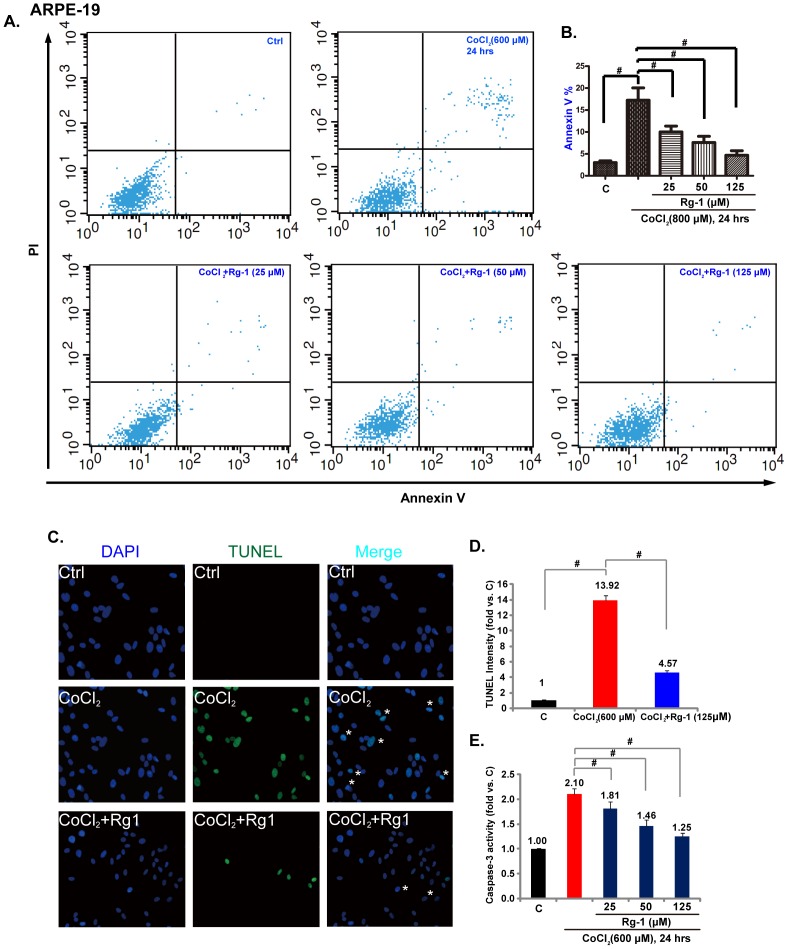
Rg-1 inhibits CoCl_2_-induced apoptosis of RPE cells. ARPE-19 cells were pre-treated with indicated concentration of Rg-1 (25, 50 and 125 µM) for 2 hrs, followed by CoCl_2_ (600 µM) administration, cells were further cultured for 24 hrs, cell apoptosis was analyzed by annexin V assay (A) and TUNEL staining (C), data of three sets of independent experiments were quantified (B for annexin V, D for TUNEL). The activity of caspase-3 was also detected (E). Data were expressed as mean±SD. *p<0.05. For C and D, Rg1's concentration was 125 µM.

The above results have shown that Rg-1 inhibits CoCl_2_-induced RPE cell damage. Next, we tested whether such an effect by Rg-1 was due to apoptosis prevention. The annexin V flow cytometry analysis was used to test the apoptosis of RPE cells. As shown in Figure 2A and B, Rg-1 pretreatment (25–125 µM) significantly inhibited RPE apoptosis by CoCl_2_ (600 µM). Note that Rg-1's anti-apoptosis effect was dose-dependent (Figure 1B).Rg-1's inhibitory role on cell apoptosis was further confirmed by TUNEL staining (Figure 2C and D) and caspase-3 activity assay (Figure 2E). Exposure to CoCl_2_ (600 µM) yielded 13.92% TUNEL-positive RPE cells (Figure 2C), and Rg-1 pretreatment (125 µM) significantly decreased the number of TUNEL-positive cells to 4.57% (Figure 2C and D). All the measurements indicated that the protective effect of Rg-1 against CoCl_2_ might be due to apoptosis inhibition.

### Rg-1 inhibites CoCl_2_-induced ROS production and MAPK activation in RPE cells

**Figure 3:**
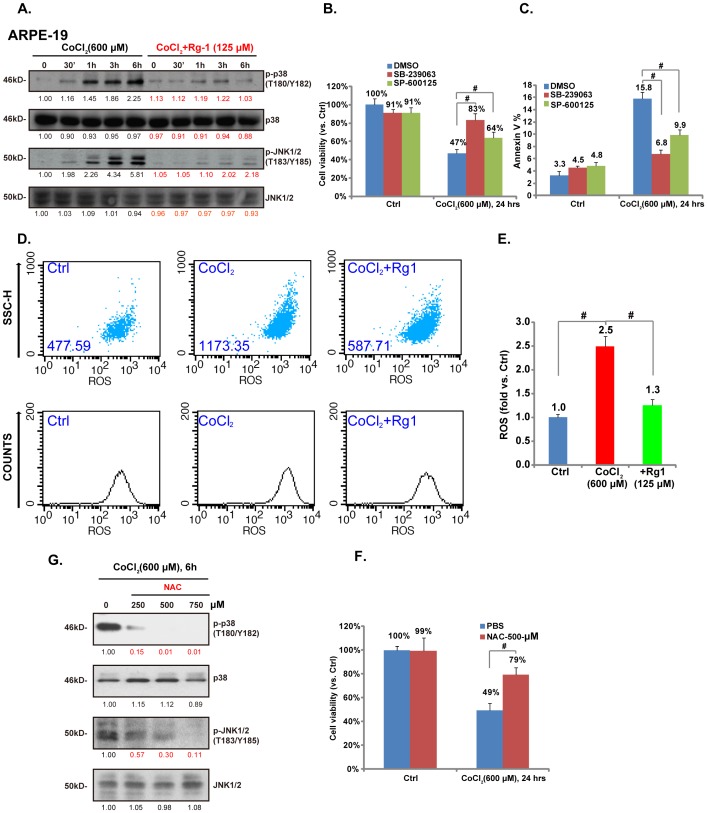
Rg-1 inhibites CoCl_2_-induced ROS production and MAPK activation in RPE cells. ARPE-19 cells were pre-treated with Rg-1 (125 µM) for 2 hrs, followed by CoCl_2_ (600 µM) administration, cells were further cultured for indicated time points, phospho (p)- and total (t)-p38 and JNK1/2 were analyzed by Western blots (A). ARPE-19 cells were pre-treated with p38 inhibitor SB-239063 (10 µM) or SP-600125 (10 µM) for 1 hr, followed by CoCl_2_ (600 µM) administration, and cell viability and apoptosis were analyzed by MTT assay (B) and annexin V flow cytometry (C) respectively 24 hrs after stimulation. Cellular ROS level was also tested (D and E). The numbers in D mean relative ROS-DCFDA signal readout. ARPE-19 cells were pre-treated with indicated anti-oxidant NAC for 2 hrs, followed by CoCl_2_ (600 µM) administration, cells were further cultured, p38 and JNK1/2 activation (F) as well as cell viability (G) were shown. Western blot data were quantified as described. Data were expressed as mean±SD. **^#^** ***p***<0.05.

Activation of the p38 and JNK MAPK pathway is strongly associated with cell apoptosis of many stresses. In our settings, we first examined the role of CoCl_2_ on p38 and JNK activation in RPE cells. Western blot results in Figure 3A demonstrated that CoCl_2_ (600 µM) administration induced significant p38 and JNK activation in RPE cells. Meanwhile, SB-239063, the p38 inhibtior and the JNK inhibitor SP-600125 supressed CoCl_2_ (600 µM)-induced RPE cell death (Figure 3B) and apoptosis (Figure 3C), suggesting that p38/JNK1/2 activation was required for CoCl_2_-induced RPE cell damage. SB-239063 led to a stronger rescue effect than SP-600125 (Figure 3B and C), indicating that p38 activation might be more important than JNK activation in mediating CoCl_2_-induced RPE cytotoxicity. Importantly, as shown in Figure 3A, Rg-1 pre-administration (125 µM, 2 hrs pretreatment) almost blocked CoCl_2_-induced JNK and p38 activation in RPE cells, suggesting that the protective role of Rg-1 against CoCl_2_ might be assocaited with JNK/p38 inhibition. Further, we also examined ROS accmulation in CoCl_2_-treatd RPE cells, and results showed that Rg-1 inhibited intracelluar cellular ROS production by CoCl_2_ (Figure 3D and E). The fact that anti-oxidatn NAC inhibited CoCl_2_-induced JNK/p38 activation (Figure 3G) and RPE cell death (Figure 3F) suggested that ROS is required for MAPK activaiton and cytotoxicity by CoCl_2_.

### Inactivation of mTORC1 by CoCl_2_ and restoring by Rg-1

**Figure 4:**
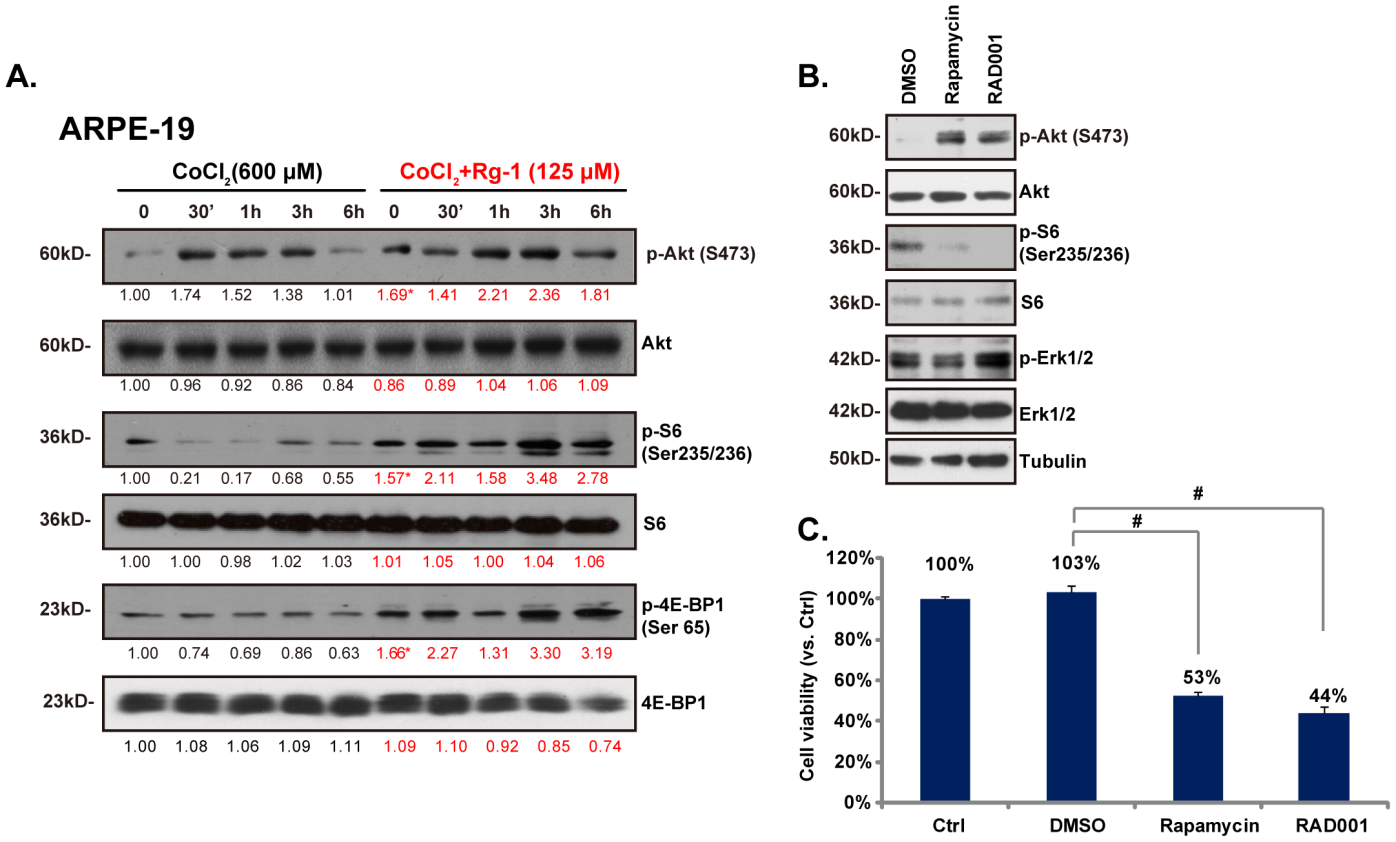
Inactivation of mTORC1 by CoCl_2_ and restoring by Rg-1. ARPE-19 cells were pre-treated with Rg-1 (125 µM) for 2 hrs, followed by CoCl_2_ (600 µM) administration, cells were further cultured for indicated time points, phospho (p)- and total -Akt, S6 and 4E-BP1 were analyzed by Western blots (A). ARPE-19 cells were treated with RAD001 (200 nM) and rapamycin (400 nM), after 4 hrs, phospho (p)- and total (t)-Akt, S6 and p-Erk1/2 as well as tubulin were examined (B), cell viability was analyzed by MTT assay after 24 hrs (C). Western blot data were quantified as described. Data were expressed as mean±SD. **^#^** ***p***<0.05.

Activation of Akt and mammalian target of rapamycin complex 1 (mTORC1) signaling plays important roles in cell survival, and apoptosis resistance. We then tested this pathway in CoCl_2_-treated RPE cells. Western blot results in Figure 4A showed that in RPE cells CoCl_2_ administration activated Akt while inhibiting mTORC1 (Figure 4A). Note that Akt activation was reflected by increased expression of phospho (p)-Akt (Ser 473), while p-S6 and p-4E-BP1 downregulation confirmed mTORC1 inhibition in CoCl_2_ treated cells(Figure 4A). Meanwhile, RAD001 and rapamycin, two mTORC1 inhibitors, blocked S6 phosphorylation (Figure 4B), both inhibitors also reduced RPE cell viability (Figure 4C). Thus mTORC1 activation is required for RPE cell survival. Importantly, Rg-1 pre-administration restored mTORC1 activation in CoCl_2_-treated RPE cells (Figure 4A). These results indicated that rescue of mTORC1 signaling by Rg-1 could also be linked to its pro-survival effect against CoCl_2_. It should be noted that Rg-1 alone also enhanced Akt and mTORC1 activaiton in RPE cells (Figure 4A, star labbelled), which might explain why we saw an increased cell survival by Rg-1 alone (Figure 1A).

### AMPK activation by CoCl_2_ mediates mTORC1 inhibition, reversed by Rg-1

**Figure 5:**
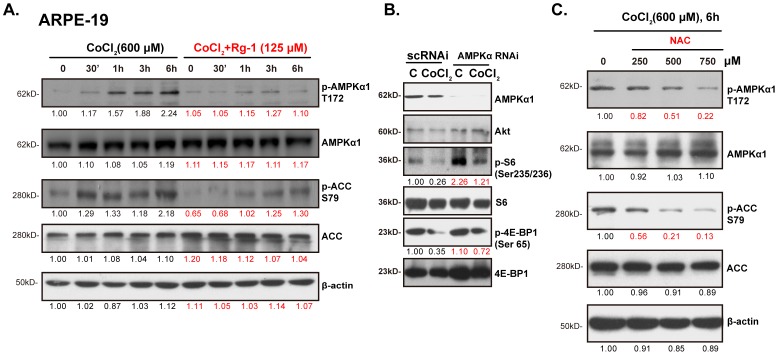
AMPK activation by CoCl_2_ mediates mTORC1 inhibition, reversed by Rg-1. ARPE-19 cells were pre-treated with Rg-1 (125 µM) for 2 hrs, followed by CoCl_2_ (600 µM) administration, cells were further cultured for indicated time points, phospho (p)- and total -AMPKα1 and ACC were tested by Western blots, β-actin was also shown (A). The scramble or AMPKα1/2 shRNA expressing stable ARPE-19 cells were stimulated with CoCl_2_ (600 µM) for 6 hrs, p- and t-S6/4EBP1, as well as AMPKα and Akt were tested (B). ARPE-19 cells were pre-treated with indicated concentration of NAC for 2 hrs, followed by CoCl_2_ (600 µM) administration, AMPK activation and β-actin expression were shown (C). Western blot data were quantified as described.

Above results showed that CoCl_2_ inhibited mTORC1 activation in RPE cells. The well-known inhibitory kinase of mTORC1 is AMP-activated protein kinase (AMPK). Activated AMPK inhibits mTORC1 through the following mechanisms: by phosphorylation and activation of TSC2 (tuberous sclerosis protein 2) [28], or by phosphorylation of Raptor (regulatory associated protein of mTOR) [29]. We then tested AMPK activation in CoCl_2_-treated RPE cells. Western blot results in Figure 5A showed that CoCl_2_ induced significantly AMPK activation (AMPK/ACC phosphorylation) in RPE cells. While Rg-1 pre-administration almost blocked AMPK activation by CoCl_2_. Significantly, CoCl_2_-induced mTORC1 inhibition was alleviated in stable RPE cells with AMPKα knockdown (Figure 5B), indicating that activation of AMPK by CoCl_2_ is required for mTORC1 inhibition in RPE cells, and Rg-1 restores mTORC1 activity probably through preventing AMPK activation. Notably, the anti-oxidant N-acetylcysteine (NAC) dose-dependently inhibited AMPK activation by CoCl_2_ (Figure 5C), which indicates that ROS might be the upstream signal for CoCl_2_-induced AMPK activation in RPE cells (see [30], [31]), and Rg-1's inhibitory effect on AMPK activation could be due to its ability on ROS scavenging (Figure 3D and E).

### Hypoxia-induced cytotoxicity and cytoprotection by Rg-1 in hypoxic RPE cells

**Figure 6:**
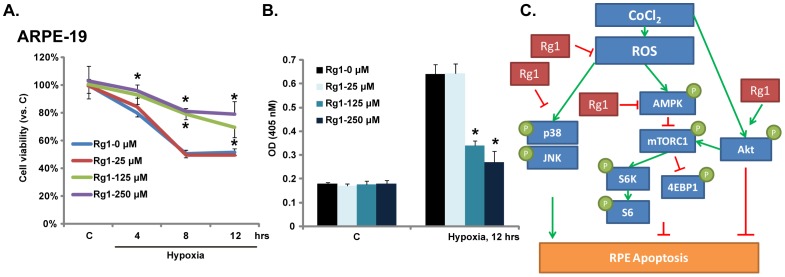
Hypoxia-induced cytotoxicity and cytoprotection of Rg-1 in hypoxic RPE Cells. ARPE-19 cells were pre-treated with Rg-1 (25, 125 and 250 µM) for 2 hrs, cells were further cultured in an anaerobic chamber for 4, 8 and 12 hrs, cell viability was then tested by MTT assay (A). ARPE-19 cells were pre-treated with Rg-1 (25, 125 and 250 µM) for 2 hrs, cells were further cultured in an anaerobic chamber for 12 hrs, Histone-DNA ELISA OD was shown (B). (C) The proposed signaling pathway of the present study: In cultured RPE cells, Rg-1 exerts a significant cytoprotective effect against damage caused by CoCl_2_ or hypoxia. CoCl_2_ induces ROS production and subsequent JNK/p38 MAPKs activation leading to RPE cell apoptosis, which are largely inhibited by Rg-1. CoCl_2_ inhibits pro-survival mTORC1 activation (S6/4E-BP1 phosphorylation) through activation of AMPK signaling, while Rg-1 inhibits AMPK activation and restores mTORC1 activation. Rg-1 also enhances cytoprotective Akt activation in RPE cells. Note that the green arrow stands for “promotes” or “activates”, while the red bar stands for “inhibits”. * ***p***<0.05 vs. the hypoxia group.

When RPE cells were cultured in an anaerobic chamber (hypoxia), cell death developed in a time-dependent manner, and cell viability OD reduced when hypoxia lasted (Figure 6A). The most significant cell death induced by hypoxia was observed after 8 hrs after anaerobic chamber incubation. Rg-1 ranging from 125–250 µM significantly protected ARPE-19 cells from hypoxia-induced damage, and cell viability was restored. 25 µM of Rg-1 had no obvious protective effect (Figure 6A). Meanwhile, cell apoptosis was tested by Histone-DNA ELISA assay. Hypoxia induced significant apoptosis in ARPE-19 cells, such an effect was largely inhibited by Rg-1 (125–250 µM) pre-incubation (Figure 6B).

## Discussion

Retinal ischemia is a pathological feature shared by many eye diseases including central retinal vein occlusion (CRVO), branch retinal vein occlusion (BRVO), diabetic retinopathy (DR), and retinopathy of prematurity (ROP). Thus, these diseases are collectively referred as ischemic retinopathies [32]. At the cellular level, ischemic retinal injury consists of a self-reinforcing destructive mechanisms involving decrease of mitochondrial enzyme activity, calcium homeostasis damage and oxidative stress caused by increased energy failure and hypoxia [1]. As such, strategies against hypoxia-induced retinol cell damage should be vital to restore the normal retinal environment and prevent retinal damage. In the current study, we found that Ginsenoside Rg-1 protects RPE cells from hypoxia or chemical hypoxia (CoCl_2_)-induced cell damage.

Ginsenoside Rg-1, which displays significant neuroprotective effect during transient ischemia, protects neurons by activating of anti-apoptotic mechanisms [19], [33]. Thus, Rg-1 should be a promising candidate to treat ischemic retinopathy. However, at least to our knowledge, there has been no investigation on the potential role of Rg-1 on retina and retinal cells. In the current study, we demonstrated that Rg-1 exerted a significant protective role against CoCl_2_ and hypoxia-induced RPE cell damage, which was associated with changes of multiple signaling pathways (Figure 6C). ROS production [34] and subsequent MAPK activation were proposed as major signaling mechanisms mediating cell death by hypoxia [35], [36], [37], and we found that Rg-1 significantly inhibited CoCl_2_-induced ROS accumulation and JNK/p38 activation by CoCl_2_ (Figure 6C). Further, CoCl_2_ inhibited pro-survival mTORC1 activation in RPE cells, and activation of AMPK was involved in the process. Rg-1 restored mTORC1 activation probably by inhibiting AMPK activation in RPE cells (Figure 6C).

Recently, different groups including us [38] have demonstrated that AMPK is an important regulator for cell apoptosis [39], [40], [41], [42], [43], [44].This serine/threonine kinase is originally thought to act as a cellular energy sensor by stimulating ATP-producing catabolic pathways and inhibiting ATP-consuming anabolic pathways [45], [46]. We and others proved that sustained AMPK activation could also promote cell apoptosis by regulating its downstream signals, including JNK [47], [48], [49], p38 [50], [51], p53 [52], [53]. Meanwhile, AMPK inhibits mTORC1 activation [54], [55], [56]. Our recent study and others have discovered that UV and H_2_O_2_-induced RPE cell apoptosis was also associated with AMPK activation [31], [38], [57]. In the current study, we also observed a significant AMPK activation in CoCl_2_ treated RPE cells, and Rg-1 pre-treatment blocked AMPK activation by CoCl_2_. The fact that NAC inhibited AMPK activation by CoCl_2_ suggests that ROS might be responsible for AMPK activation by CoCl_2_, and Rg-1's inhibition on AMPK might be due to its role as an ROS scavenger.

Interestingly, although CoCl_2_ inhibited mTORC1 activation in RPE cells, it simultaneously activated Akt. These results suggested that CoCl_2_-mediated mTORC1 inhibition was not dependent on its effect on Akt, rather Akt activation by CoCl_2_ could be a negative feedback effect after mTORC1 inhibition. (see in other studies [58]). As a matter of fact, both RAD001 and rapamycin, two mTORC1 blockers, also activated Akt (Ser 473 phosphorylation) in RPE cells (Figure 4B). These results suggested that CoCl_2_ mediated mTORC1 inhibition was probably due to AMPK activation, but not Akt. In conclusion, we here demonstrated the significant cytoprotective ability of Rg-1 against hypoxia- and CoCl_2_-induced cytotoxicity in RPE cells, such an effect by Rg-1 was associated with ROS-MAPK inhibition and AMPK/mTOR regulation in RPE cells. Our results indicated that Rg-1 could be further investigated as a novel cell-protective agent for retinal ischemia.

## Materials and Methods

### Cell culture

Human retinal pigment epithelial cells (ARPE-19 cell line), a gift from Dr. Fu Shang at Tufts University [59], [60], were maintained in Dulbecco's Modified Eagle's Medium(DMEM)/Nutrient Mixture F-12 (DMEM/F12, Gibco Life Technologies, Carlsbad, CA), supplemented with 10% fetal bovine serum (FBS) (Hyclone, Shanghai, China), penicillin/streptomycin (1:100, Sigma, St. Louis, MO), and 4 mM L-glutamine and 0.19% HEPES (Sigma), in a humidified incubator at 37°C and 5% CO_2_.

### Reagents and chemicals

Ginsenoside Rg-1 (purity 99%) was purchased from Amresco (Solon, OH). Cobalt chloride (CoCl_2_) was supplied as a sterile ready-to-use 800 mM stock solution (Sigma, France). SP-600125 (the JNK inhibitor), SB-239063 (the p38 inhibitor), N-acetylcysteine (NAC), rapamycin and RAD001 were purchased from Calbiochem (Darmstadt, Germany). All phosphorylated kinase antibodies and their non-phosphorylated control antibodies were obtained from Cell Signaling Tech (Danvers, MA). Rabbit mono-clonal antibodies against tubulin and β-actin were purchased from Sigma (St. Louis, MO).

### Cell viability assay

RPE cell viability was measured by the 3-[4,5-dimethylthylthiazol-2-yl]-2,5 diphenyltetrazolium bromide (MTT) method as described [38]. Briefly, RPE cells were collected and seeded in 96-well plate at a density of 2×10^5^ cells/cm^2^. Different seeding densities were optimized at the beginning of the experiments. After overnight incubation, cells were exposed to indicated reagents at 37°C. After treatment, 20 µl of MTT tetrazolium (Sigma, St. Louis, MO) salt dissolved in Hank's balanced salt solution at a concentration of 5 mg/ml was added to each well. Twenty-four hrs later, the medium was aspirated carefully from each well, and 150 µl of DMSO (Sigma, St. Louis, MO) was added to dissolve formazan crystals, and the absorbance of each well was obtained using a plate reader at a test wavelength of 490 nm with a reference wavelength of 630 nm. Media-only treated cells served to indicate 100% cell viability, and the relative survival was defined as absorbance of treated wells divided by that of controls. For each treatment, 6-wells were included. All experiments were performed in triplicate.

### TUNEL staining

As reported [38], RPE cell apoptosis was detected by the TUNEL (Terminal deoxynucleotidyl transferase dUTP nick end labeling) In Situ Cell Death Detection Kit (Roche Molecular Biochemicals, Indianapolis, IN, USA) according to the manufacturer's instructions. RPE cells were also stained with 4′,6′-diamino-2-phenylin-dole(DAPI, blue fluorescence; Molecular Probes) to visualize the cell nuclei. RPE cell apoptosis was determined by TUNEL fluorescence intensity recorded by the confocal microscopy. All experiments were performed in triplicate.

### Caspase-3 activity assay

After treatment, the caspase-3 activity of RPE cells was measured by a fluorometric caspase-3 assay kit (Kai-Ji, Nanjing, China) according the manufacturer's instructions. Briefly, the RPE cells were collected and lysed using lysis buffer provided. The caspase-3 activity colorimetric assay is based on the hydrolysis of the peptide substrate acetyl-Asp-Glu-Val-Asp- p-nitoaniline (Ac-DEVD-pNA) by caspase-3, resulting in the release of the p-nitroaniline (pNA) moiety. pNA has a high absorbance at 405 nm, which was detected by the plate reader. Caspase-3 activity in treatment group was normalized to control group. All experiments were performed in triplicate.

### Cell apoptosis detection by annexin V staining

RPE cell apoptosis was detected by the Annexin V Apoptosis Detection Kit (Beyotime, Shanghai, China) according to the manufacturer's protocol. Briefly, after treatment, one million RPE cells were stained with propidium iodide (PI) solution (5mg/mL, Invitrogen, CA) and annexin V (1mg/mL, Invitrogen, CA) for 30 min in 37°C. Both early (annexin V^+^/PI^−^) and late (annexin V^+^/PI^+^) apoptotic cells were sorted by fluorescence-activated cell sorting (FACS) (Becton Dickinson FACS Calibur). All experiments were performed in triplicate.

### Apoptosis assay by enzyme-linked immunosorbent assay (ELISA)

As previously reported [61], the Cell Apoptosis Histone-DNA ELISA Detection Kit PLUS (Roche, Palo Alto, CA) was utilized to further test quantify cell apoptosis, according to the manufacturer's protocol. Briefly, RPE cells were collected and seeded in 96-well plate at a density of 2×10^5^ cells/cm^2^. After treatment, the cytoplasmic histone/DNA fragments from RPE cells were extracted and bound to the immobilized anti-histone antibody (included in the kit). Subsequently, the peroxidase-conjugated anti-DNA antibody (included in the kit) was added for the detection of immobilized histone/DNA fragments. After addition of substrate for peroxidase, the spectrophotometric absorbance of the samples was determined using a plate reader at a test wavelength of 405 nm. All experiments were performed in triplicate.

### Reactive oxygen species (ROS) assay

The ROS level was determined by carboxy-H2DCFDA staining assay, which is based on the fact that the nonpolar, nonionic H2-DCFDA crosses cell membranes and is hydrolyzed into non-fluorescent H2-DCF by intracellular esterase. In the presence of ROS, H2-DCF is rapidly oxidized to become highly fluorescent DCF. After treatment, ARPE-19 cells were incubated with 1 µM of carboxy-H2-DCFDA at 37°C for 30 min. Cells (1*10^6^) were then resuspended in phosphate-buffered saline (PBS, pH 7.4) and sent to flow cytometry analysis (BD bioscience). The percent of fluorescence-positive cells was recorded on a spectofluotometer using excitation and emission filters of 488 and 530 nm. All experiments were performed in triplicate.

### Stable siRNA knockdown of AMPKα1/2

The lentiviral particles containing scramble or AMPKα1/2 shRNAs[62] were purchased from Santa Cruz Biotech (Santa Cruz, CA), lentiviral shRNAs were added to the ARPE-19 cells for 36 hrs, and stable clones expressing corresponding shRNAs were selected by puromycin (1.0 µg/ml). Cell culture medium containing puromycin was renewed every 48 hrs, until resistant colonies can be identified (4–5 passages). The expression level of AMPKα1/2 and loading controls in stable cells were tested.

### Western blot analysis and data quantification

After indicated treatment, aliquots of 30 µg of lysed proteins (lysed by 40 mM HEPES [pH 7.5], 120 mM NaCl, 1 mM EDTA, 10 mM pyrophosphate, 10 mM glycerophosphate, 50 mM NaF, 0.5 mM orthovanadate, EDTA-free protease inhibitors [Roche] and 1% Triton) from each sample were separated by 10% SDS polyacrylamide gel electrophoresis and transferred onto a polyvinylidene difluoride (PVDF) membrane (Millipore, Bedford, MA). After blocking with 10% instant non-fat dry milk for 1 hr, membranes were incubated with specific antibodies overnight at 4°C followed by incubation with secondary antibodies for 45 minutes at room temperature. The Western blot results were visualized by ECL machine. The intensity of each blot was quantified using ImageJ software after normalization to corresponding loading controls, and their value was expressed as fold change vs. the band labeled with “1.00”. All experiments were performed in triplicate.

### Hypoxia-Induced Cytotoxicity in RPE cells

4×10^5^ of ARPE-19 were seeded onto 96-well microplates in a final volume of 100 µL culture medium per well. Hypoxia-related cytotoxicity over time was investigated by placing the cells in an anaerobic chamber (<1% oxygen) for 4, 8, and 12 hrs. Part of wells were pretreated with indicated concentration of Rg-1, at the indicated time points, an MTT assay was performed. All experiments were performed in triplicate.

### Statistical analysis

Individual culture dishes or wells were analyzed separately (no pooling of samples was used). In each experiment a minimum of three wells/dishes of each treatment were used. All the values in the figures are expressed as the means ± SD. Statistical significance was determined using one-way ANOVA followed by a Scheffe's f-test by using SPSS software (SPSS Inc., Chicago, IL, USA). ***p***<0.05 was considered to be significant.
