# Expression of Concern: Ginsenoside Rg-1 Protects Retinal Pigment Epithelium (RPE) Cells from Cobalt Chloride (CoCl_2_) and Hypoxia Assaults

**DOI:** 10.1371/journal.pone.0304598

**Published:** 2024-05-24

**Authors:** The PLOS ONE Editors

After this article [1] was published, concerns were raised about results presented in Figs 2, 3 and 5. Specifically:

- In Fig 2C, the DAPI Ctrl, DAPI CoCl_2_, Merge Ctrl and Merge CoCl_2_ panels appear similar.
- The p-p38 panel in Fig 3A and the p-AMPKα1 T172 panel in Fig 5A appear similar when dimensions are adjusted.

**Fig 2:**
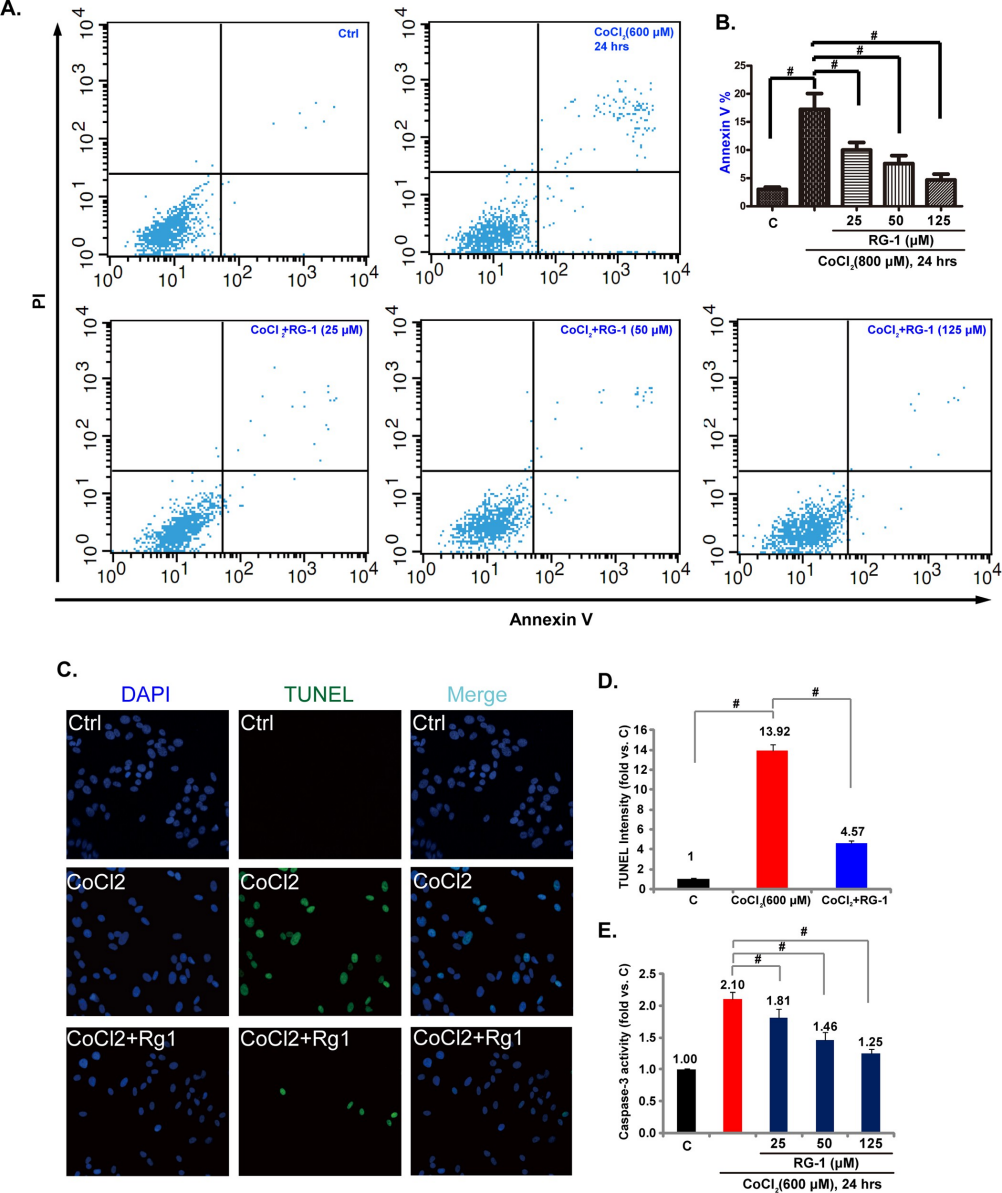
Rg-1 inhibits CoCl_2_-induced apoptosis of RPE cells. ARPE-19 cells were pre-treated with indicated concentration of Rg-1 (25, 50 and 125 μM) for 2 hrs, followed by CoCl_2_ (600 μM) administration, cells were further cultured for 24 hrs, cell apoptosis was analyzed by annexin V assay (A) and TUNEL staining (C), data of three sets of independent experiments were quantified (B for annexin V, D for TUNEL). The activity of caspase-3 was also detected (E). Data were expressed as mean ± SD. *p < 0.05. For C and D, Rg1’s concentration was 125 μM.

In response to queries about the experiments in Fig 2C, the corresponding author stated that an error occurred in preparing the DAPI Ctrl and Merge Ctrl panels. A corrected version of Fig 2 is provided here where the three Ctrl panels in Fig 2C have been replaced. The underlying images for Fig 2C are in S1 File. The *PLOS ONE* Editors consider this concern resolved.

In response to queries about the experiments in Figs 3A and 5A, the corresponding author stated that the p-p38 panel in Fig 3A and the p-AMPKα1 T172 panel in Fig 5A are different and that the original image files underlying these panels are no longer available. The *PLOS ONE* Editors therefore consider this concern to be unresolved.

Individual-level data and original uncropped image files for the charts and panels in Fig 2B–2D are provided here in S1 File. The corresponding author stated that the remainder of the data underlying this article [1] are no longer available.

In light of the unresolved concerns in Figs 3A and 5A, and the unavailability of the original underlying data for these figures, the *PLOS ONE* Editors issue this Expression of Concern.

In addition, please note that reference 57 [2] in this article [1] was withdrawn [3] before this article [1] was published.

## Supporting information

S1 File Underlying data in support of Fig 2B–2E including individual-level quantitative data for Fig 2B, 2D and 2E. Fig 2B, 2D and 2E are based on all images (sets 1–3). Fig 2C shows set 1 only.
(PDF)

## References

[1] Li K-r Zhang Z-q, Yao J Zhao Y-x, Duan J, et al. (2013) Ginsenoside Rg-1 Protects Retinal Pigment Epithelium (RPE) Cells from Cobalt Chloride (CoCl_2_) and Hypoxia Assaults. PLoS ONE 8(12): e84171. doi: 10.1371/journal.pone.0084171 24386346 PMC3873980

[2] Cao Cong, Lu Shan, Kivlin Rebecca, Wallin Brittany, Card Elizabeth, Bagdasarian Andrew, Tamakloe Tyrone, Chu Wen-ming, Guan Kun-liang, Wan Yinsheng, AMP-activated Protein Kinase Contributes to UV- and H_2_O_2_-induced Apoptosis in Human Skin Keratinocytes*, Journal of Biological Chemistry, Volume 283, Issue 43, 2008, Pages 28897–28908, ISSN 0021-9258, 10.1074/jbc.M804144200 18715874 PMC2570892

[3] Cao Cong, Lu Shan, Kivlin Rebecca, Wallin Brittany, Card Elizabeth, Bagdasarian Andrew, Tamakloe Tyrone, Chu Wen-ming, Guan Kun-liang, Wan Yinsheng, Withdrawal: AMP-activated protein kinase contributes to UV- and H_2_O_2_-induced apoptosis in human skin keratinocytes., Journal of Biological Chemistry, Volume 285, Issue 19, 2010, Page 14842, ISSN 0021-9258, 10.1074/jbc.A109.804144 29874310 PMC2863220

