# Supplementary material for: Expression of Concern: Ginsenoside Rg-1 Protects Retinal Pigment Epithelium (RPE) Cells from Cobalt Chloride (CoCl2) and Hypoxia Assaults
Source: PLoS One. 2024 May 24;19(5):e0304598. doi: 10.1371/journal.pone.0304598 (PMC11125501; doi:10.1371/journal.pone.0304598)

SET1 (Repeat-1)

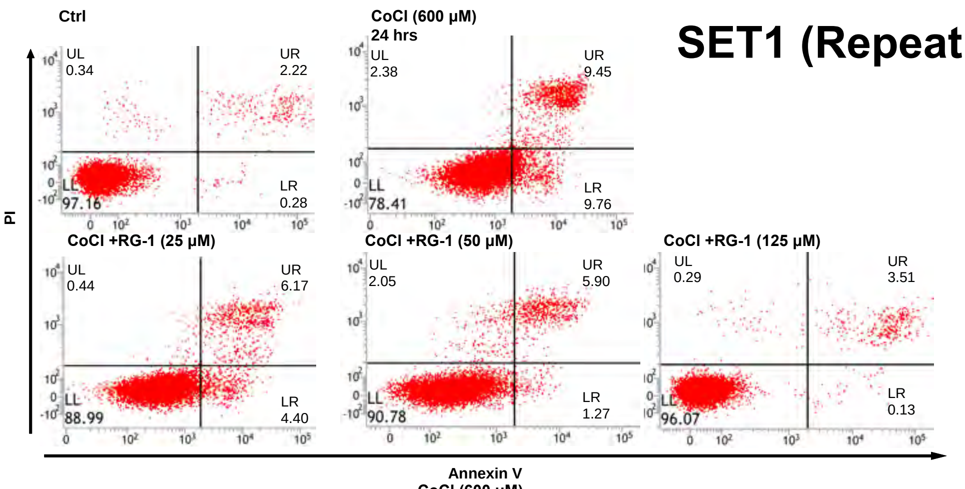

SET2 (for presentation)

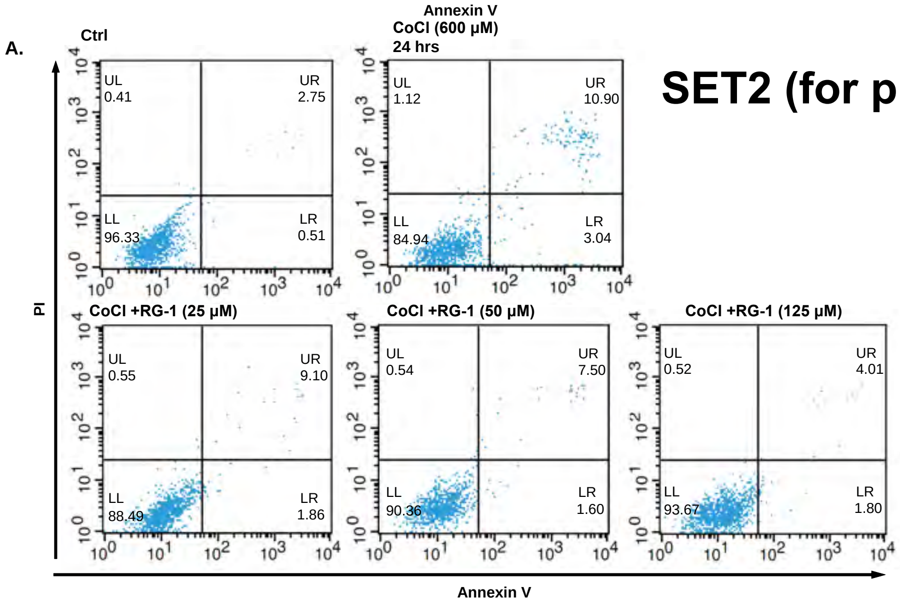

SET3 (Repeat-2)

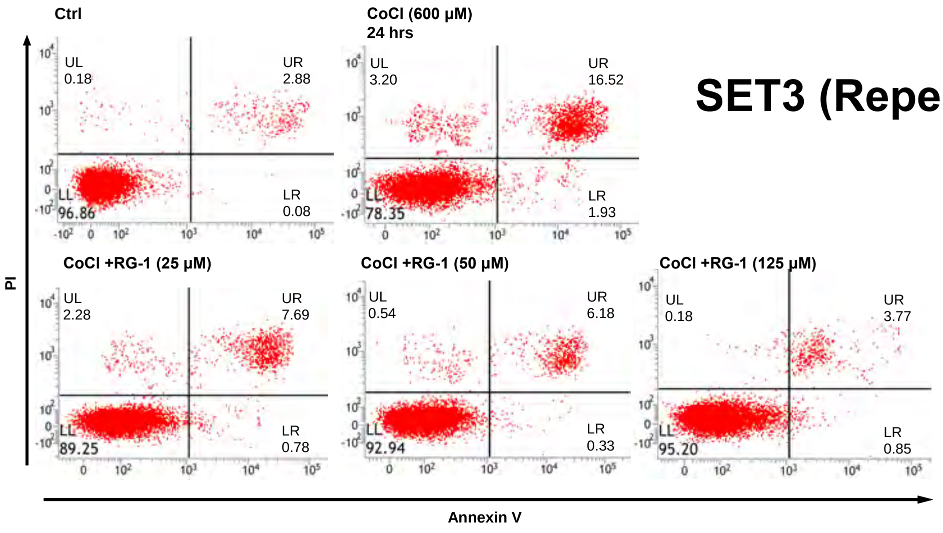

Together

Annexin V%					
SET 1	CTRL	CoCl (600 µM	RG-1 (25 µM)	RG-1 (50 µM)	RG-1 (125 µ
EARLY APOPTOSIS	2.22	9.45	6.17	5.9	3.51
LATE APOPTOSIS	0.28	9.76	4.4	1.27	0.13
Together	2.5	19.21	10.57	7.17	3.64
SET 2					
EARLY APOPTOSIS	2.75	10.9	9.1	7.5	4.01
LATE APOPTOSIS	0.51	3.04	1.86	1.6	1.8
Together	3.26	13.94	10.96	9.1	5.81
SET 3					
EARLY APOPTOSIS	2.88	16.52	7.69	6.18	3.77
LATE APOPTOSIS	0.08	1.93	0.78	0.33	0.85
Together	2.96	18.45	8.47	6.51	4.62
Average	2.90	17.2	10.0	7.59	4.69
SD	0.38	2.84	1.33	1.34	1.08

Bar graph

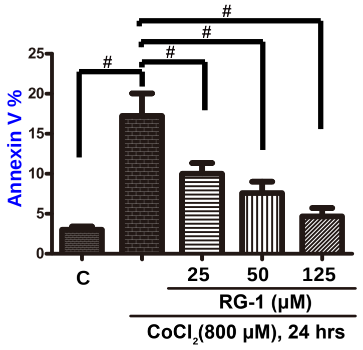

SET1 (For data presentation in Figure 2C)

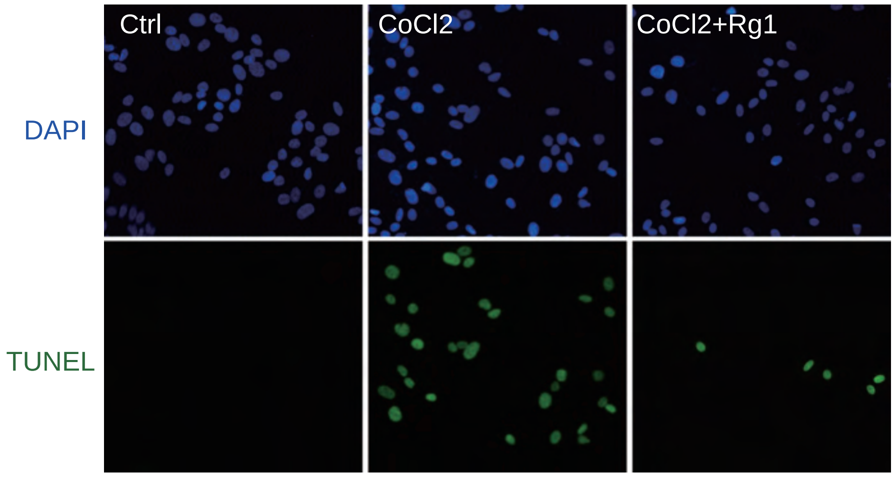

SET2

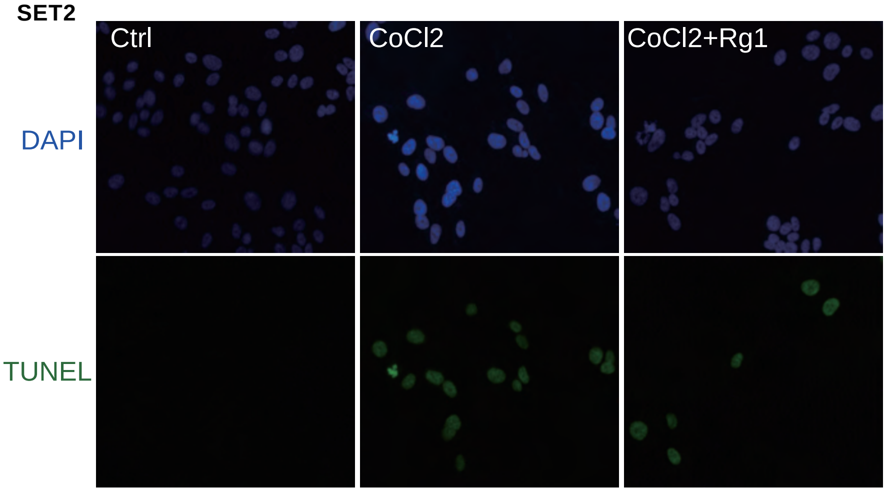

SET3

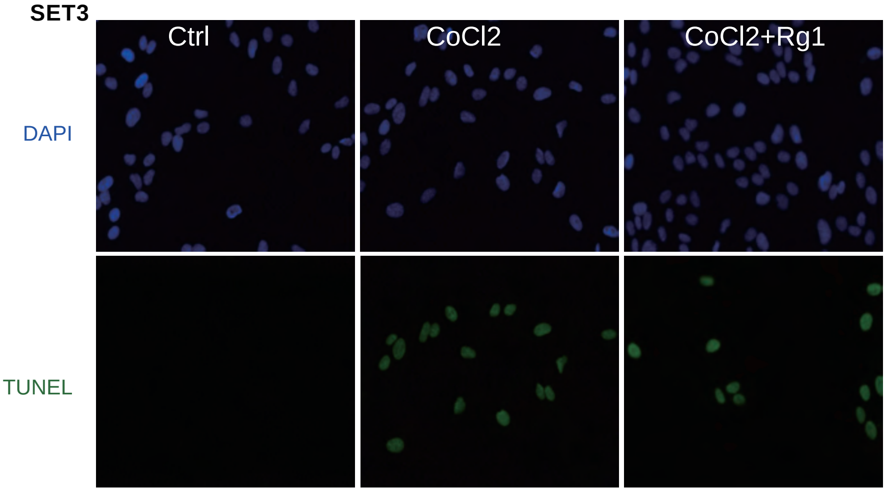

TUNEL Intensity value

SET1	View1	View2	View3	View4	View5	Average	Fold vs C
CTRL	0.035	0.035	0.035	0.034	0.035	0.035	1.000
CoCl (600 μ	0.483	0.477	0.426	0.464	0.474	0.465	13.359
Cocl+Rg1	0.163	0.224	0.160	0.164	0.155	0.173	4.980
SET2	View1	View2	View3	View4	View5	Average	
CTRL	0.025	0.025	0.025	0.025	0.025	0.025	1.000
CoCl (600 μ	0.364	0.342	0.351	0.382	0.376	0.363	14.583
Cocl+Rg1	0.114	0.112	0.091	0.128	0.117	0.113	4.521
SET2	View1	View2	View3	View4	View5	Average	
CTRL	0.042	0.042	0.042	0.041	0.041	0.042	1.000
CoCl (600 μ	0.578	0.571	0.582	0.553	0.592	0.575	13.814
Cocl+Rg1	0.187	0.130	0.195	0.179	0.187	0.176	4.219
						Together	SD
						1.00	0.00
						13.92	0.62
						4.57	0.38

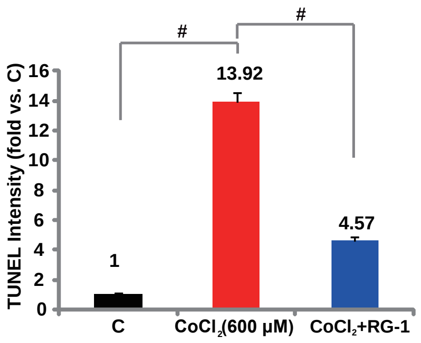

Caspase-3 activity OD

SET 1	CTRL	CoCl (600 µM	RG-1 (25 µM	RG-1 (50 µM	RG-1 (125 µM
OD VALUE	0.231	0.513	0.451	0.367	0.305
FOLD VS Ctrl	1	2.219512	1.95122	1.585366	1.317073
SET 1	CTRL	CoCl (600 µM	RG-1 (25 µM	RG-1 (50 µM	RG-1 (125 µM
OD VALUE	0.195	0.387	0.335	0.267	0.229
FOLD VS Ctrl	1	1.980769	1.716154	1.364617	1.173077
SET 3	CTRL	CoCl (600 µM	RG-1 (25 µM	RG-1 (50 µM	RG-1 (125 µM
OD VALUE	0.188	0.396	0.329	0.270	0.235
FOLD VS Ctrl	1	2.1102	1.754864	1.437861	1.251474
Average	1	2.10	1.81	1.46	1.25
SD	0	0.12	0.13	0.11	0.07

E.

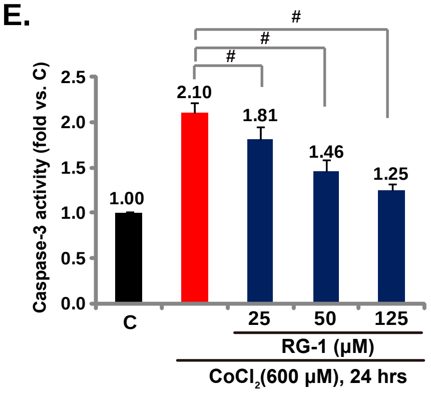

Supplement: S1 File — Fig 2B, 2D and 2E are based on all images (sets 1–3). Fig 2C shows set 1 only. (PDF) [file pone.0304598.s001.pdf]
